# Canine babesiosis treatment rates in South African veterinary clinics between 2011 and 2016

**DOI:** 10.1186/s13071-018-2962-x

**Published:** 2018-07-03

**Authors:** Robert Lavan, Kaan Tunceli, Hendrik de Swardt, Carolyn Chelchinskey, Mats Abatzidis, Rob Armstrong

**Affiliations:** 1 Outcomes Research, Animal Health, Center for Observational and Real-World Evidence, Merck & Co., Inc, Kenilworth, NJ USA; 2 Otomys Software Solutions CC, PO Box 904 287, Faerie Glen, 0043 South Africa; 3 MSD Animal Health, 20 Spartan Road, Spartan, Kempton Park, 1619 South Africa; 4 MSD Animal Health, 2 Giralda Farms, Madison, NJ 07940 USA

**Keywords:** Acaricide, *Babesia*, Dog, Veterinary practice, Healthcare use rate

## Abstract

**Background:**

South African veterinarians report the perception of a multi-year decline in the number of dogs presenting with clinical babesiosis, a common and serious disease of dogs in the country. This study tested this observation through analysis of veterinary hospital medical records from 2011 through 2016.

**Methods:**

Medical records were collected from 44 participating South African veterinary hospitals. The collected medical records were searched to enumerate the number of *Babesia*-specific medication treatments administered to dogs at all participating hospitals. A healthcare use rate was calculated for canine babesiosis treatment for each calendar year from 2011 to 2016. The healthcare use rate numerator was the total number of canine babesiosis treatments and the denominator was the total dog visits to all participating veterinary practices over the same period.

**Results:**

There were 2.6 million dog visits to 44 participating veterinary practices between 2011 and 2016. The number of canine babesiosis treatments for each year in chronological order starting with 2011 was: 2957; 2679; 2456; 2746; 2272; and 1592. South African regions with the highest number of canine babesiosis treatments were Gauteng, Free State and Mpumalanga. The overall calculated healthcare use rate for canine babesiosis treatment declined 72% over the study period from 1.18% in 2011 to 0.33% in 2016. The steepest decline of 31% was observed between 2015 and 2016.

**Conclusions:**

South African veterinary practices saw a decline in canine babesiosis treatment administration from 2011 to 2016 with the steepest decline beginning in 2015.

## Background

Dogs in South Africa are at risk for a severe form of canine babesiosis also called “biliary fever” that clinically resembles human *Plasmodium falciparum* malaria [1, 2] with the highest incidence in summer [1, 3, 4]. The virulent parasite *Babesia rossi* is considered a primary cause of canine babesiosis in South Africa [1] with jackals as reservoir host [5]. *Babesia canis* has distinguishable subgroups with different levels of virulence and PCR-based survey work also found the less virulent *Babesia vogeli* widely distributed in South Africa [6]. Cases of canine babesiosis with additional complications - including cerebral effects, enterorrhagia, hemoconcentration, acute renal failure and/or pulmonary edema - are considered common by South African veterinarians and death can occur in more than 10% of cases [1, 7].

*Babesia* infection follows transmission of the causative agent to a dog during attachment and feeding by an infected tick, usually either *Haemaphysalis elliptica* or *Rhipicephalus sanguineus* [1, 8]. It can take more than 24 hours of attachment to transmit the parasite from tick to dog and this time delay provides a window of opportunity to kill the tick and prevent transmission [9].

The incubation period between the tick bite and the onset of clinical signs can be as short as two weeks [10] and signs are the result of intravascular red blood cell destruction following intra-erythrocytic parasite asexual reproduction [1, 7, 8, 10–13]. Clinical signs can include fever, pallor, anemia, icterus and hematuria leading to weakness and respiratory difficulty secondary to blood loss [1, 10–13], although infected dogs may not develop any signs and may remain subclinically infected [12]. Clinically affected dogs presented at veterinary hospitals are typicallly diagnosed through visualization of intracellular parasites on a stained blood smear [1, 7, 8, 10–12].

Veterinarians treat canine babesiosis in South Africa with antiparasitic drug administration as well as blood transfusions and supportive care [1, 8, 10–12]. Medications used to treat canine babesiosis in South Africa, like diminazene aceturate or imidocarb diproprionate, are used exclusively for this purpose in dogs, although they may have other label claims (e.g. *Theileria* and *Anaplasma*) in other species [7, 13]. The standard treatment protocol for dogs is to administer injections of one of these medications once or twice over a 14 day period. Therefore, counting the number of doses of these drugs admininstered to dogs provides a method to estimate the healthcare use rate in veterinary clinics and to detect changes in canine babesiosis risk.

Veterinary hospitals use practice management software to store their transaction and medical records. These medical records can be filtered, searched and downloaded, allowing patient and owner confidentiality to be maintained by replacing patient identity information with a unique ID number. The software captures data on all hospital patients and procedures including records on diagnoses and treatments. The inclusion of treatments in the record means that the number of babesiosis medication doses administered and the dates of administration to dogs can be readily found.

Veterinarians practicing in South Africa report a perceived decline in the presentation of dogs with clinical babesiosis over the past few years. The objective of this study was to examine trends in canine babesiosis treatment in South African veterinary practices based on healthcare use analysis of data from veterinary practice clinical records. Healthcare use analysis can be readily calculated from medical record data and estimates the relative proportion of medical resources allocated to a particular condition, such as canine babesiosis.

## Methods

All veterinary hospitals in South Africa that currently use a proprietary medical records management system (Microvet, Otomys Software Solutions) were invited to partipate in this records review study, as long as the hospital met specific inclusion criteria. These criteria were that the hospital: gave signed permission to complete a medical records search and download; operated in a region where canine babesiosis treatment is administered; and, maintained data for at least one full year in the 2011–2016 study period. Baseline data were obtained for each participating study hospital including: practice name, practice address, and number of canine patient visits at the hospital for each year in the time period 2011–2016.

The overall study population includes all dogs brought to participating veterinary clinics during the six year study period, from 2011 to 2016 inclusive. A data search algorithm was prepared and used to complete a retrospective search of all medical records during the study period to identify specific transactions. This algorithm specifically identified and extracted the records of babesiosis treatments (i.e. medications prescribed to treat canine babesiosis) administered to dogs. Initially, a limited search was completed in five veterinary practices to identify records of dogs treated with diminizine aceturate or imidocarb diproprionate, and/or with a *Babesia*-positive blood smear and/or an assigned babesiosis diagnosis keyword. This limited search found that the administered babesiosis treatment record was the optimal method for case identification in the database. Medical records of treated dogs included the diagnostic keyword 75% of the time and a record of a confirmatory babesiosis blood test 10% of the time.

Positive canine babesiosis cases were identified by searching for records that include prescription and/or administration of specific medications exclusively used in dogs to treat babesiosis in South Africa. The dates of drug administration for an individual dog were compared, to identify treatment for a single infection (treatments within 30 days of each other) and to recognize re-infection treatments (more than 30 days apart). The goal was to determine the number of times a veterinary hospital treated canine babesiosis in a given year. A healthcare use rate for canine babesiosis treatment administration was calculated for each year between 2011 and 2016. An individual veterinary clinic rate was calculated by dividing the total number of canine babesiosis treatments administered during the year by the total number of canine visits to the clinic during the year. This information was summed across regions and then overall for the South African cohort. The total number of canine visits for the year includes the summed total of veterinary records of all dogs visiting the veterinary hospital for any reason (healthy and sick dog visits) over each 12 month (calendar year) period.

## Results

**Fig. 1:**
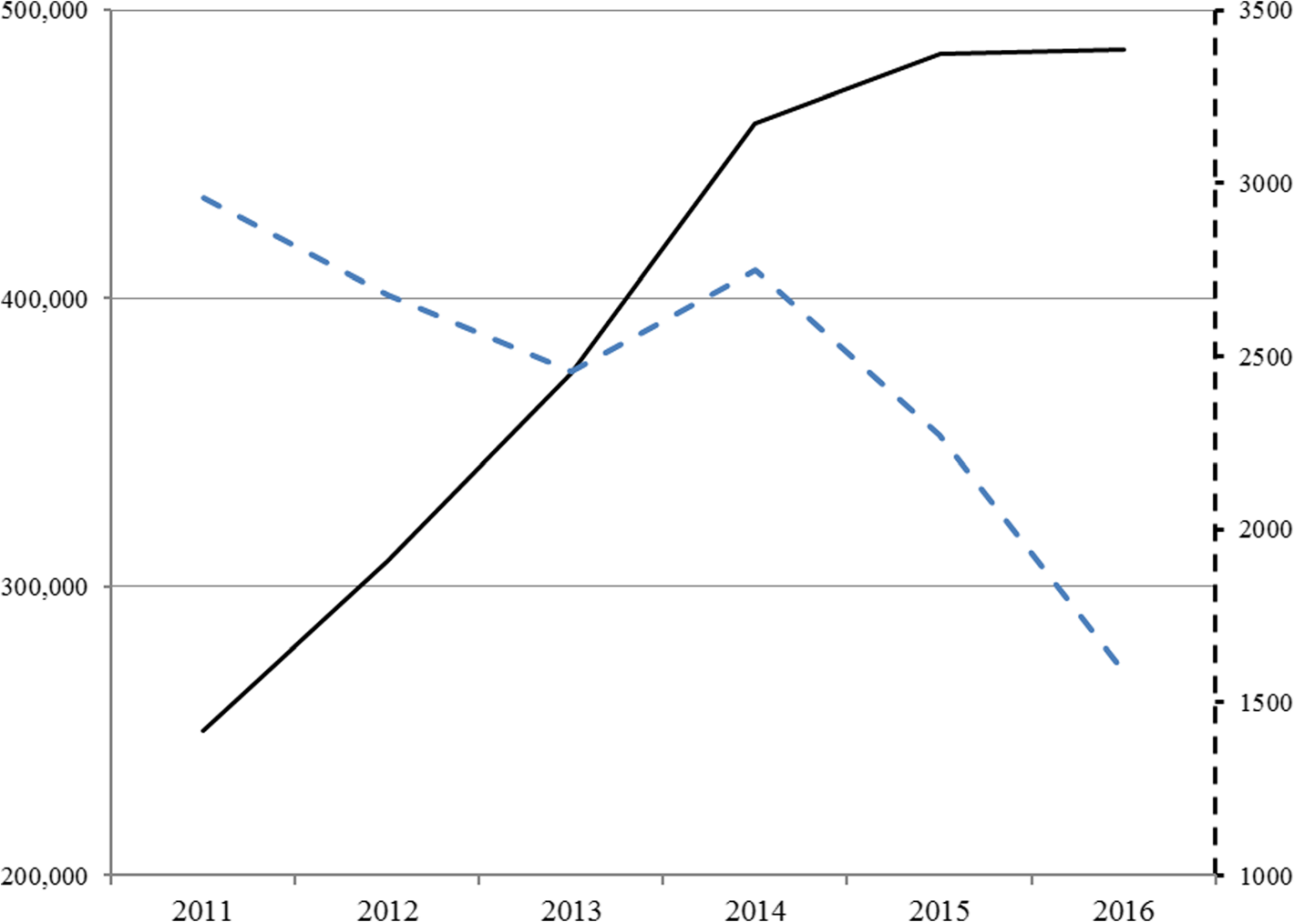
Number of dog visits (left axis) and canine babesiosis treatments (right axis, dashed line) at 44 veterinary hospitals in South Africa between 2011 and 2016

**Fig. 2:**
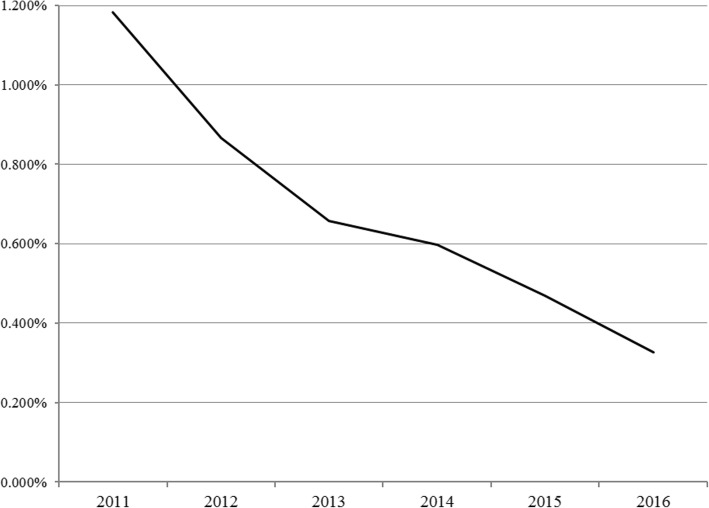
Healthcare use rate for canine babesiosis treatment at 44 South African veterinary hospitals between 2011 and 2016

Forty-four veterinary hospitals across South Africa (Table 1) met the eligibility conditions and were enrolled in the study as participants. The number of canine babesiosis treatments administered varied across the country, with three regions (Gauteng, Free State and Mpumalanga) having the most *Babesia* treatments administered over the 6-year study period (Table 1). The total number of dog visits at veterinary hospitals (Fig. 1) increased by 94.4% over the study period with the steepest rise from 2011 to 2014 (84.1%), a slower rise between 2014 and 2015 (5.2%) and then essentially flat from 2015 to 2016 (0.4%, Table 2). The absolute number of canine babesiosis treatments administered (Fig. 1) declined by 46% over the study period, with an initial shallow decrease from 2011 to 2013; an increase of 11.8% in treatments from 2013 to 2014 (Table 2) and then a steep decline from 2014 to 2016. The healthcare use rate for canine babesiosis treatment declined (Fig. 2) by 72% over the 6-year study period with the most rapid decline between 2015 and 2016 (Table 2).

**Table 1:** Number of canine babesiosis treatments (per clinic mean) administered in veterinary hospitals in South Africa by year and by hospital region

Region	Participating hospitals	2011	2012	2013	2014	2015	2016	6 year per clinic average^a^
Gauteng	18	120 (13)	95 (14)	69 (17)	87 (16)	68 (17)	44 (17)	81
Free State	3	175 (3)	138 (3)	97 (3)	88 (3)	86 (3)	76 (3)	110
Mpumulanga	6	86 (4)	95 (4)	114 (5)	106 (6)	89 (6)	69 (6)	93
Western Cape	10	31 (7)	19 (8)	21 (10)	24 (10)	19 (10)	11 (10)	21
Northwest Province	1	57 (1)	37 (1)	30 (1)	35 (1)	28 (1)	7 (1)	32
KwaZulu Natal	1	50 (1)	47 (1)	40 (1)	43 (1)	30 (1)	23 (1)	39
Eastern Cape	2	13 (2)	29 (2)	41 (2)	32 (2)	15 (2)	15 (2)	24
Limpopo	2	12 (2)	45 (2)	25 (2)	26 (2)	16 (2)	14 (2)	23
Northern Cape	1	18 (1)	7 (1)	12 (1)	21 (1)	14 (1)	13 (1)	14
Overall Average		83 (34)	71 (36)	58 (42)	65 (42)	53 (43)	37 (43)	

^a^An unweighted mathematical average calculated from the sum of each yearly average divided by 6

**Table 2:** Proportional change from prior year and overall six-year change for canine babesiosis treatments, total dog visits and canine babesiosis treatment healthcare use ratio at veterinary hospitals in South Africa

Study years	Year-to-year change in no. of dogs treated for babesiosis (%)	Year-to-year change in total dog visits (%)	Year-to-year change in healthcare use rate (%)
2011–2012	-9.4	+23.6	-28.0
2012–2013	-8.3	+21.0	-23.5
2013–2014	+11.8	+23.1	-6.2
2014–2015	-17.3	+5.2	-21.3
2015–2016	-29.9	+0.4	-31.2
Overall 2011–2016	-46.2	+94.4	-72.0

## Discussion

The results of this study confirm the perception of clinical veterinarians in South Africa that the healthcare use rate for veterinary services to treat canine babesiosis declined, dropping by72% between 2011 and 2016 (Table 2 and Fig. 2). The healthcare use rate declined most rapidly (30.2%) between 2015 and 2016 (Table 2). The records search methodology used in this study has limitations that could lead to an underestimation of the number of *Babesia*-infected dogs if these dogs were not treated at a veterinary clinic; to an overestimation of infected dogs if false positive cases are included; or with a potential sampling bias if practices that use the software system are also consistently likely to under- or over-diagnose. Changes in diagnostic techniques are not expected to have resulted in the decrease in reported canine babesiosis cases over the study period and blood smear evaluation continues to be the diagnostic method of choice for South African companion animal veterinarians in private practice. Future improvements in diagnostic techniques such as PCR used in association with improved treatment options could further reduce the incidence of canine babesiosis.

The canine babesiosis healthcare use rate is a reflection of both the number of canine *Babesia* cases seen at veterinary hospitals and the total number of dog visits to the same hospitals. Either a decrease in the number of canine babesiosis treatments (the numerator) and/or an increase in the number of veterinary hospital dog office visit (the denominator) would lead to a reduction in the healthcare use rate. Results from this study suggest that in the period from 2011 to 2014, the decrease in the healthcare use rate for canine babesiosis was affected by the rapid increase in the number of canine office visits (Fig. 1) and the relatively modest variations (± 10%) in canine babesiosis treatment rate (Fig. 1). The increase in canine office visits may be driven by socioeconomic factors associated with growth in the dog owning population and the increasing personal wealth of dog owners. However, during the period between 2014 and 2016, the number of canine visits to South African veterinary hospitals in this study were growing at single digit rates or were essentially flat, while the number of canine babesia cases were falling by 20–30% per year (Table 2). Therefore, during the 2014–2016 period, annual reductions in the healthcare use rate appear to be more affected by the annual reduction in the number of treatments administered than by growth in the number of dogs visiting the hospital (Fig. 1).

The decrease in the number of dogs being treated for canine babesiosis in these South African veterinary hospitals in the 2014–2016 period could be explained by: a decrease in the number of dogs with clinical disease secondary to increased host resistance; reduced pathogen virulence; or vector challenge reduction. With reference to the third factor, a new class of acaricidal molecules, the isoxazolines, was introduced for dogs in South Africa in 2014, and might have contributed to the trends observed. There were no other new classes of acaricidal products for dogs introduced during this period, although other acaricidal products may reduce the risk of canine babesiosis transmission [11]. The increased rate of dog presentation to veterinary hospitals over the study period (Fig. 1) may have been associated with better preventive medicine, including possible increased use of acaricidal protection in general and increased adherence seen with convenient options for acaricide administration [14] for dog owners. Isoxazoline treatments completely prevented canine babesiosis transmission in challenge studies [9, 15] suggesting that treated dogs will have protection from this disease in the field. The steepest annual declines in the canine babesiosis healthcare use rate observed in this study occurred after these treatments were introduced in South Africa in 2014 (Table 2). However, data limitations associated with the methodology and the descriptive nature of this study do not permit a specific causal determination.

## Conclusions

South African veterinary practices saw a decline in canine babesiosis treatment administration from 2011 to 2016 with the steepest decline seen between 2015 and 2016.

## Abbreviation

PCR: Polymerase chain reaction
